# Neuroangiostrongyliasis: Rat Lungworm Invades Europe

**DOI:** 10.4269/ajtmh.22-0782

**Published:** 2023-02-20

**Authors:** Claudia Paredes-Esquivel, Pilar Foronda, Claire Panosian Dunavan, Robert H. Cowie

**Affiliations:** University of the Balearic Islands Palma, Spain E-mail: claudia.paredes@uib.es; Institute of Tropical Diseases and Public Health of the Canary Islands Tenerife, Spain E-mail: pforonda@ull.edu.es; David Geffen School of Medicine University of California Los Angeles Los Angeles, California E-mail: cpanosian@mednet.ucla.edu; Pacific Biosciences Research Center University of Hawaii Honolulu, Hawaii E-mail: cowie@hawaii.edu

Dear Editor,

A recent short review in this journal^1^ on the global spread of the rat lungworm, *Angiostrongylus cantonensis* (Nematoda: Angiostrongylidae), the cause of neuroangiostrongyliasis (NAS), noted that it had been reported recently in the Canary Islands^2^^,^^3^ and Mallorca.^4^ Both of these are Spanish territories, the former in the Atlantic Ocean off northwest Africa and the latter in the Mediterranean Sea, off the coast of Spain. In light of these reports, continental Europe was considered threatened by this important zoonotic parasite.^1^

Confirming this concern, the parasite has now been detected in the city of Valencia on the Spanish mainland.^5^ Among 27 rats trapped, 29 adult *A. cantonensis* were found in four, from locations both near the port and several kilometers inland. This is the first report of *A. cantonensis* in continental Europe.

Globally, *A. cantonensis* is the primary etiological agent causing eosinophilic meningitis. It was first discovered in 1933 in southern China and has now spread widely, primarily across the tropics and subtropics, as reflected in the distribution of human cases of NAS, which can be life threatening.^1^ The only known autochthonous case of NAS in Europe was diagnosed in Paris in 2016; however, the source of this infection was never identified, as the patient had not traveled outside of France since the 1980 s other than to Morocco more than 2 years previously, and reported no consumption of possible hosts.^6^ Otherwise, all cases diagnosed in Europe have been in travelers returning from endemic areas.^7^^,^^8^

This report of *A. cantonensis* in Valencia indicates that the parasite is probably established in the city; accordingly, additional surveys to determine how far it has spread should be undertaken in the surrounding region, both in rats (definitive hosts) and snails (intermediate hosts) as well as in various paratenic and accidental hosts such as the hedgehogs in which *A. cantonensis* was found in Mallorca.^4^ Despite the parasite’s lack of tolerance of cold temperatures,^9^ which explains why it has largely been confined to tropical and subtropical regions in the past, with a foothold in Europe it could spread farther across the continent, potentially to more temperate regions, as has already occurred in Australia^10^ and the United States.^11^ Furthermore, as the climate warms, even more northern parts of Europe may become accessible to *A. cantonensis*, as seen in China.^9^

It is therefore imperative that medical practitioners in Europe become more aware of this parasite and the diagnosis and treatment of the uncommon but potentially fatal disease it causes. Educating the general public on how to avoid contracting NAS—namely, by not eating raw snails or slugs; keeping a close eye on toddlers and children playing in gardens, who may put snails or slugs in their mouths; thoroughly inspecting and washing fresh produce, especially leafy green vegetables; and controlling populations of rats and snails/slugs—is equally important. And, finally, as this new report suggests, epidemiological and parasitological surveys and screening efforts in Europe should now pay special heed to detecting *A. cantonensis*.
